# Manchester Meeting

**Published:** 1902-08-02

**Authors:** 


### The British Medical Association.

### MANCHESTER MEETING.

The annual meeting of the British Medical Association opened on July 29th under the presidency of Mr. Walter Whitehead, F.R.C.S.Edin., consulting surgeon to the Royal Infirmary. The central meeting place was at the Owens College, where, on Monday evening, a considerable number of members of the Association had already put in an appearance. Several tents have been erected in the College quadrangle, and it may be mentioned as somewhat of a novelty at these meetings that the annual dinner is this year being given under canvas. The meeting was, as is usual, commenced by religious services, one being held at the Cathedral, where Bishop Thornton delivered an eloquent sermon, and another at the Roman Catholic Church of the Holy Name, where the preacher was the Rev. Charles Coupe.

The first general meeting was held on Tuesday afternoon, when the chair was taken by Dr. G. Bagot Ferguson, of Cheltenham, the retiring President, who, after referring to the events of the past year, introduced Mr. Whitehead, saying that he wished him every success and satisfaction in his honourable office. He felt sure that the same help and assistance so freely accorded to himself would be also extended to their new President.

Mr. Whitehead was warmly received on taking the chair, and after various loyal and other resolutions, Mr. Andrew Clark, the treasurer, proposed that the financial statement for the year ending December 31, 1901, together with the estimate for revenue and expenditure for 1903, be received and adopted.

Dr. J. Brassey Brierley raised various difficulties and objections, but in the end the resolution was carried.

On Wednesday the various sections commenced work. In the sections of Medicine, Surgery, and Obstetric Medicine the stated discussions were opened. In Ophthalmology, Dr. Edridge Green proposed the following resolution:—"That in the opinion of this section the employment of the Holmgren Test for Colour Blindness by the Board of Trade is most unsatisfactory, as the inefficient character of this test is now well known." On being put to the vote, however, the resolution was lost.

In the section of Navy, Army, and Ambulance, a discussion on the Treatment of Wounded in Naval Actions was opened by Fleet-Surgeon Gilbert Kirker, M.D., R.N., while in that on Tropical Diseases, one on the Prophylaxis and Treatment of Beri-Beri was opened by Dr. Patrick Manson, C.M.G.

